# Outcomes of Vitrectomy for Macular Pathologies Associated with Idiopathic Vasoproliferative Retinal Tumor

**DOI:** 10.3390/jcm14176221

**Published:** 2025-09-03

**Authors:** Masatoshi Shinozaki, Hiromi Ohara, Tomohiko Torikai, Takashi Koto, Makoto Inoue

**Affiliations:** Kyorin Eye Center, Kyorin University School of Medicine, 6-20-2 Shinkawa, Mitaka 186-8611, Tokyo, Japan; shinozaki@ks.kyorin-u.ac.jp (M.S.); hiromi-ohara@ks.kyorin-u.ac.jp (H.O.); tomohiko-torikai@ks.kyorin-u.ac.jp (T.T.); koto@ks.kyorin-u.ac.jp (T.K.)

**Keywords:** vasoproriferative tumor, epiretinal membrane, macular hole, vitrectomy

## Abstract

**Background/Objectives:** A vasoproliferative retinal tumor (VPT) is a rare, benign, vascular tumor that is found in the peripheral retina. We retrospectively investigated the clinical characteristics and the outcomes of pars plana vitrectomy (PPV) on idiopathic VPTs. **Methods:** The medical records of 13 eyes of 12 patients who underwent PPV for an idiopathic VPT were reviewed. The chief complaint of the patients, the location and size of the tumor, the presence of concomitant retinal disorders, the treatment procedures, and the best-corrected visual acuity (BCVA) were evaluated. **Results:** The mean age was 51.2 years, with 4 men and 8 women. The chief complaints at the initial visit were metamorphopsia in 6 eyes, decreased vision in 6 eyes, and blurry vision in 1 eye. A hemangioma was detected in the inferior-temporal quadrant in 11 eyes and in the superior-temporal quadrant in 2 eyes. An epiretinal membrane was present in all 13 eyes with tractional and exudative retinal detachment in 4 eyes, macular hole in 2 eyes, and vitreous hemorrhage in 3 eyes. During the vitrectomy, a posterior vitreous detachment was created in 10 eyes (77%), photocoagulation was performed for the hemangioma in all 13 eyes, cryotherapy in 4 eyes, and direct diathermy in 2 eyes. The visual acuity improved significantly from 0.51 ± 0.66 logMAR units at the baseline to 0.05 ± 0.21 logMAR units at the final examination (*p* = 0.002). **Conclusions:** Vitrectomy for idiopathic VPTs is effective in improving the visual acuity. Idiopathic VPTs are often associated with epiretinal membranes, and patients present with various types of visual disorders.

## 1. Introduction

A retinal vasoproliferative tumor (VPT) is a rare, acquired retinal hemangioma, and it has only recently been classified as a distinct clinical entity [1,2,3]. However, it used to be called by various names. In 1982, Baines et al. [4] reported peripheral telangiectatic nodules in eyes with subretinal fluid, yellow exudates, and an epiretinal membrane (ERM) at the posterior pole. Laqua and Wessing [5] reported a unilateral localized peripheral vascular lesion with macular pucker or cystoid macular edema. Shields et al. [6] reported on 12 patients who had a unilateral, solitary, vascular mass located in the sensory retina. These masses were reported to be acquired retinal hemangiomas. In 1995, Shields et al. [2] reported on the clinical manifestations of 103 patients with an acquired retinal vascular tumor and named this clinical entity as VPTs, and this name is still accepted today.

Shields et al. classified VPTs into idiopathic or primary VPT (74%) and secondary ocular disorder VPT (26%) [2,3]. Idiopathic VPTs are usually solitary lesions (87%) in contrast to secondary tumors that are often multiple (42%) [2]. Most cases of VPTs are unilateral, but bilateral cases have been reported, especially in patients with secondary lesions. Shields and associates reported the clinical characteristics of 219 cases of idiopathic VPT, whose mean age was 46 years [2]. Sixty-one percent of their patients were women, and the hemangiomas were located anterior to the equator in 78% of their cases [2]. They reported that 66% of the hemangiomas were located in the inferior-temporal quadrant, 14% in the superior-temporal quadrant, and 20% in other quadrants [2]. The decimal visual acuity was ≥0.4 in 66%, 0.1 to 0.3 in 19%, and <0.1 in 15% [2].

VPTs are sporadic and are diagnosed at any age although the majority are detected in the third and fourth decades of life [2,3]. The associated vitreoretinal findings include intraretinal exudation, secondary exudative retinal detachments, vitreous cells, vitreous hemorrhage, ERM, and macular edema [2]. Secondary VPTs are associated with other ocular disorders including intermediate uveitis [2,3,7,8], retinitis pigmentosa [2,3,9], toxoplasmosis [2], toxocariasis [2], retinal detachment [2], retinochoroidal coloboma [2], Coats disease [10,11], retinopathy of prematurity [12], neurofibromatosis [13], aniridia [14], and Waardenburg’s syndrome [15].

Small, asymptomatic peripheral VPTs lacking significant exudation or macular involvement can be managed by periodic observations [1]. Laser photocoagulation, transconjunctival cryopexy, or intravitreal injection of anti-vascular endothelial growth factor (anti-VEGF) agents has been used to treat VPTs with a significant number of exudations or with a retinal detachment [1,16,17,18,19]. Larger lesions have been successfully treated with plaque radiotherapy [1,20,21].

Because idiopathic VPTs are relatively rare, they are often diagnosed after they become symptomatic due to an ERM or macular edema caused by peripheral vascular lesions. We report the clinical characteristics of 13 eyes with a VPT, and the results of pars plana vitrectomy (PPV).

## 2. Materials and Methods

This single-center, observational study was approved by the Institutional Review Committee of the Kyorin University School of Medicine (863-02). It adhered to the tenets of the Declaration of Helsinki. All of the patients received a detailed explanation of the surgical and ophthalmic procedures, and all signed an informed consent form. All of the patients consented to our review of their medical records and their anonymized use in medical publications.

### 2.1. Study Population

We reviewed the medical records of 13 eyes of 12 patients that were diagnosed with an idiopathic VPT. All of the patients were examined in our clinic between October 2013 to March 2024. The mean age of the patients was 51.2 years with a range of 22 to 72 years. There were 4 men and 8 women. The mean follow-up period was 28.7 months with a range of 12 to 121 months. We determined the chief complaint at the first visit, location and size of the tumor, and any concomitant disorders of the retina at the initial examination, treatment method, and the best-corrected visual acuity (BCVA) at baseline, postoperative 1, 3, 6 months, and final examinations. The location of the VPT was in one or more of the four quadrants of the retina. We used 25-gauge or 27-gauge pars plana vitrectomy (PPV) to treat all eyes. Fundus photographs, fluorescein angiography, optical coherence tomographic (OCT) images (Spectralis^®^, Heidelberg Engineering, Heidelberg, Germany) were evaluated.

### 2.2. Surgical Techniques

All patients underwent 25-gauge (G) or 27G PPV (Constellation^®^ Vision System; Alcon Laboratories Inc, Fort Worth, TX, USA) with a widefield viewing system (Resight^®^; Carl Zeiss Meditec, Oberkochen, Germany) under topical and sub-Tenon’s anesthesia. The PPV and phacoemulsification with implantation of intraocular lens were performed in patients older than 50-years-of-age. Forty milligrams of triamcinolone acetonide (TA; MaQaid^®^; Wakamoto Pharmaceutical Co., Ltd., Tokyo, Japan) was mixed with 2.0 mL of balanced salt solution to produce the TA suspension. The TA suspension was injected intravitreally to make the vitreous cortex more visible, and a posterior vitreous detachment (PVD) was created as far peripherally as possible if it was not present. The internal limiting membrane (ILM) was peeled off with the aid of brilliant blue G staining. Laser photocoagulation was performed around the VPT, diathermy was applied directedly on the VPT, or transscleral cryopexy was applied. Fluid/air exchange was performed on 2 eyes with a macular hole (MH), and the patients were instructed to maintain a face-down position for one to two days.

### 2.3. Statistical Analyses

The decimal BCVA was converted to the logarithm of the minimum angle of resolution (logMAR) for the statistical analyses. Poorer visual acuities were graded as: counting fingers, 2.0 logMAR units; hand motion, 2.3 logMAR units; light perception, 3.0 logMAR, and no light perception, 4.0 logMAR. The baseline BCVA was compared to the BCVA at the final examination. The significance of the differences in the findings in 2 groups was determined by the Wilcoxon signed rank tests using SPSS (version 28.0; IBM, Armonk, New York, NY, USA). The 95% confidence interval (CI) of the difference was calculated with Microsoft Excel for Mac (ver. 16.99.2).

## 3. Results

### 3.1. Preoperative Demographics and Surgical Procedures

The chief complaint of the patients at the initial examination was metamorphopsia in 6 eyes (46%), decreased vision in 6 eyes (46%), and blurry vision in 1 eye (8%, Table 1). Ophthalmoscopic examinations detected a hemangioma in the inferior-temporal quadrant in 11 eyes (85%) and in the superior-temporal quadrant in 2 eyes (15%). An ERM was present in all 13 eyes with concomitant tractional exudative retinal detachment in 7 eyes (54%), MH in 2 eyes (15%), and vitreous hemorrhage in 3 eyes (23%). None of the eyes had preoperative treatments, including photocoagulation, cryopexy, and intravitreal injection of anti-VEGF agents.

**Figure 1:**
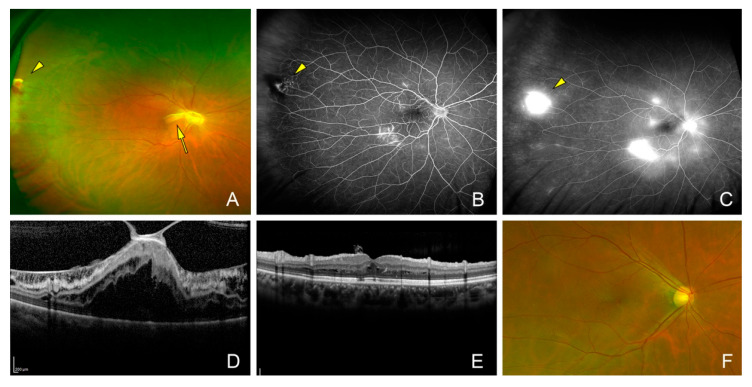
Fundus and optical coherence tomographic (OCT) images of the eye of a 35-year-old woman (Case 11). (**A**) Fundus photograph of an eye with a vasoproliferative tumor (VPT, arrowhead) and an associated epiretinal membrane (ERM, arrow). Her preoperative decimal visual acuity was 0.02. (**B**) Fluorescein angiography in the early phase and (**C**) the late phase reveals leakage from the VPT and leakage by the traction of ERM. (**D**) Cross-sectional OCT image indicating vitreomacular traction with ERM. (**E**) Cross-sectional OCT image and (**F**) fundus photograph showing resolution of the macular retinoschisis one year after the vitrectomy with inverted internal limiting membrane peeling. Vision recovered to 0.2.

**Figure 2:**
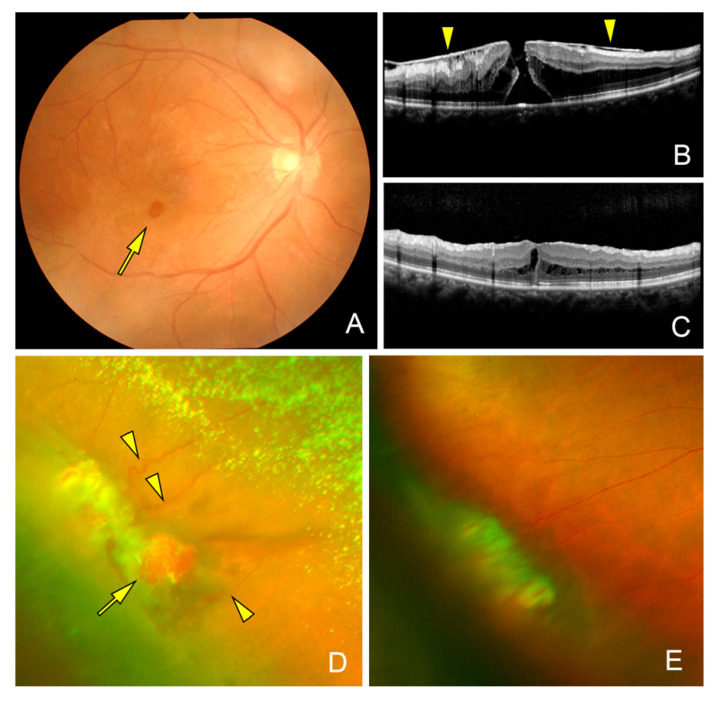
Fundus and optical coherence tomographic (OCT) images of the eye of a 53-year-old woman (Case 4). (**A**). Fundus photograph showing a macular hole (MH, arrow) and an epiretinal membrane (ERM). The ERM is attached to the edge of the MH and to the optic disk resembling a vitreomacular traction syndrome. This patient had a history of a retinal hemangioma in the right eye which was diagnosed as a vasoproliferative tumor for ten years and vitreous hemorrhages occurred three times and then disappeared spontaneously. She was referred to our clinic when she developed a MH, and her preoperative decimal visual acuity was 0.8. (**B**). Preoperative vertical OCT image showing a MH and an ERM (arrowheads) attached to the edge of the MH. (**C**). Postoperative OCT image showing a closed MH with reduction in the intraretinal cyst after vitrectomy with removal of the ERM and the internal limiting membrane. The vision recovered to 1.0 at postoperative 3 months. (**D**). Preoperative wide-angle photograph shows retinal hemangioma (arrow) surrounded by dilated retinal vessels (arrowheads), exudative changes, and asteroid hyalosis in the inferior-temporal quadrant. (**E**). Postoperative wide-angle photograph shows regressed retinal hemangioma and chorioretinal scar after direct diathermy and endolaser photocoagulation of the hemangioma.

**Figure 3:**
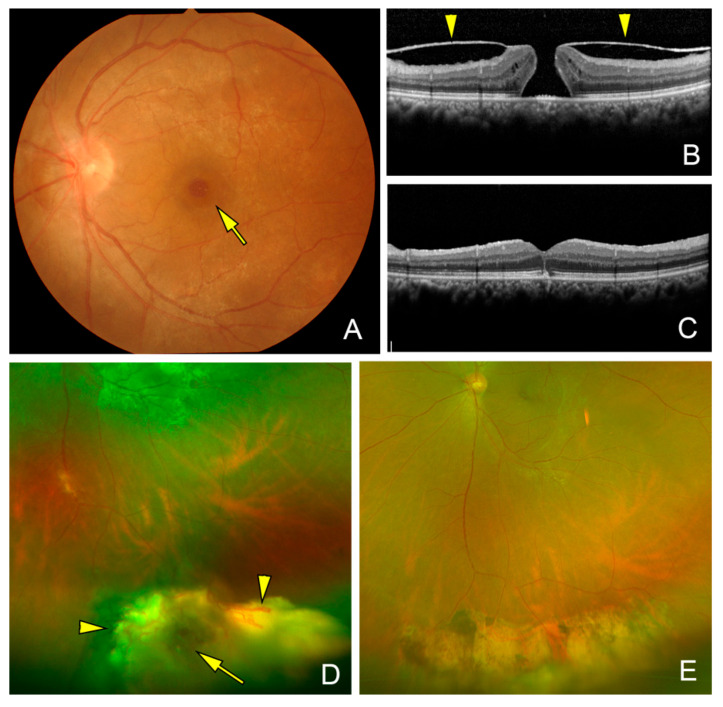
Fundus photograph and optical coherence tomographic (OCT) images of the eye of a 52-year-old woman (Case 3). (**A**). Fundus photograph shows a macular hole (MH, arrow) and an epiretinal membrane (ERM). The ERM is attached to the edge of the MH and optic disk similar to that in eyes with vitreomacular traction syndrome. This patient had a history of a retinal hemangioma in the right eye that was diagnosed as vasoproliferative tumor ten years earlier. Vitreous hemorrhages occurred three times that disappeared spontaneously. She was referred to our clinic when she developed a MH and the preoperative decimal visual acuity was 0.4. (**B**). Preoperative vertical OCT image showing a MH and an ERM (arrowheads) attached to the edge of the MH. (**C**). Postoperative OCT image shows a closed MH after vitrectomy with removal of the ERM and internal limiting membrane. The decimal BCVA recovered to 0.7 at 2 years after surgery. (**D**). Preoperative wide-angle fundus photograph showing a retinal hemangioma (arrow) surrounded by dilated retinal vessels (arrowheads) and exudative changes in the inferior quadrant. (**E**). Postoperative wide-angle photograph shows regressed retinal hemangioma and chorioretinal scar after cryotherapy of the hemangioma.

A PVD was detected in 3 eyes (23%) complicated with an ERM and absent in 10 eyes (77%, Figure 1). Two of 3 eyes with a PVD had vitreous opacities with a spontaneously detached ERM. Two eyes were complicated by a MH that was associated with an ERM (Figure 2 and Figure 3). During the vitrectomy, a PVD was created in 10 eyes, photocoagulation was performed for the hemangioma lesions in all 13 eyes, cryotherapy photocoagulation on 4 eyes (31%), and direct diathermy on 2 eyes (15%, Table 2). All of the eyes were phakic, and combined cataract surgery was performed on 8 eyes (62%).

### 3.2. Postoperative Outcomes After Vitrectomy

The BCVA improved significantly from a mean of 0.51 ± 0.66 logMAR units, 95% CI: 0.11, 0.90 at the baseline to 0.05 ± 0.21 logMAR, 95% CI: −0.07, 0.18 at the last examination (*p* = 0.002, Wilcoxon signed rank tests). The VPTs were observed as scar lesions at the last visit and did not recur during the follow-up period.

## 4. Discussion

Our study included only idiopathic VPTs, and all were treated with PPV. The mean age of our patients was 51.2 years with eight women (66.7)%. The hemangiomas were located anterior to the equator in all 13 eyes (100%), and in the inferior-temporal quadrant in 11 eyes (85%), and the decimal BCVA was >0.4 in 61.5% at the baseline. These characteristics of our patients are comparable to those reported by Shields et al. [2].

On the other hand, an ERM was associated with the VPT as the retinal lesions in 100% of our cases, which is higher than the 71% reported by Shields et al. [2]. In addition, the main symptom was metamorphopsia in 46%, decreased vision in 46%, and blurry vision in 1 eye. We found that symptomatic ERM was more frequently associated with VPT than reported by Shields et al. [2]. Therefore, these results differ from those reported by Shields et al. [2], maybe because the study was a retrospective review of all cases with diagnosed acquired retinal hemangioma or VPT, and in our study many of our VPT patients were referred to our clinic for diagnosis and treatment.

Poole Perry and associates [22] reported on the histopathological findings following enucleation in 4 cases. They reported that the VPT lesions were composed of glial cells with a scarcity of blood vessels. Initially, the VPT appeared as a pink mass in the sensory retina fed by a retinal artery and drained by a retinal vein, often with yellow intraretinal and subretinal exudations [2,3,23]. Since no histological analysis has been reported for early untreated cases [21,24], VPT is considered not to be a true neoplasm, but rather an elaborate retinal neovascularization probably induced by excessive VEGFs [2,3,23]. The VEGFs induced the lesion to assume a tumorous appearance [2,3,23]. A VPT is a reactive vascular mass without gliosis in the early stage, and the astrocytic proliferation occurs at a late stage [2,3,23]. Thus, laser, cryopexy, irradiation, and anti-VEGF agents are expected to induce vascular regression and gliosis [23]. The exudative changes induced by VEGF lead to tractional proliferations, including an ERM, and this may require vitreous surgery due to the high incidence of macular pathologies associated with ERMs [1,2,3,16,25].

The macular pathologies, including ERM, MH, cystoid macular edema, and proliferative vitreoretinopathy have been successfully treated by vitreous surgery [25,26,27,28,29,30,31]. Jong and associates reported that PPV for eyes with an epiretinal membrane and vitreous hemorrhage had a good visual outcome [26]. Zhang and associates [28] reported significant visual improvements after vitrectomy combined with episcleral cryopexy for macular complications including ERM, MH, and macular edema in eyes with a VPT. However, Garcia-Arumi and associates described a lack of significant visual improvement in 31 eyes, including 17 complicated eyes after PPV, even after successful anatomical improvement [29]. Earlier surgical intervention of PPV may be considered.

We performed vitrectomy on 13 eyes of 12 patients, and a significant improvement in the BCVA was observed in all eyes. All of the patients had an ERM with peripheral exudative changes around the VPT lesions. The MH was due to tractional proliferations by associated ERM, which were enhanced by the exudative properties of VEGF. These eyes underwent intraoperative ERM and ILM peeling to remove the tractional proliferations.

To treat a hemangioma, we used a combination of retinal photocoagulation, cryotherapy, and direct diathermy. All of the hemangiomas regressed without any recurrences. We conclude that vitrectomy is helpful in the treatment for an ERM associated with VPT, and appropriate coagulation methods can lead to the regression of the hemangioma.

There are limitations in this study. The small sample size of 13 eyes and the lack of complete uniformity in surgical techniques among the cases are limitations. The case series was limited to symptomatic eyes that had undergone vitrectomy, and asymptomatic eyes without macular involvement were not included. Additionally, this was a retrospective study, so selection bias cannot be ruled out. To validate the efficacy of vitrectomy, additional studies with a larger number of cases are needed.

## 5. Conclusions

In conclusion, idiopathic VPTs are frequently associated with ERMs, and patients often present with metamorphopsia or decreased vision. Our findings suggest that pars plana vitrectomy, combined with membrane peeling and appropriate coagulation methods, can achieve favorable anatomical and functional outcomes in selected symptomatic cases. However, given the retrospective design, small sample size, and heterogeneity of surgical procedures, these results should be interpreted with caution. Larger, prospective studies are warranted further to clarify the optimal management strategies for idiopathic VPTs.

## Figures and Tables

**Table 1:** Characteristics of patients with idiopathic vasoproliferative tumors.

Case	Side	Age	Sex	Symptom	Location of Tumor	Size of Tumor (DD)	Extent of Exudation (DD)	PVD	MH	ERM	VH	Exudative RD	Tractional RD
1	L	22	F	decreased VA	temporal-inferior	1	2	−	−	+	−	−	+
2	R	51	F	metamorphopsia	temporal-inferior	2	8	−	−	+	−	−	−
3	L	52	F	metamorphopsia	temporal-inferior	3	7	−	+	+	−	−	−
4	R	53	F	decreased VA	temporal-inferior	2	4	−	+	+	−	−	−
5	R	35	F	blurry vision	temporal-inferior	2	4	−	−	+	−	−	+
6	L	72	F	metamorphopsia	temporal-inferior	6	8	+	−	+	+	+	−
7	R	36	F	metamorphopsia	temporal-superior	3	7	−	−	+	−	−	−
8	R	72	M	decreased VA	temporal-inferior	1	3	−	−	+	−	+	−
8	L	72	M	decreased VA	temporal-inferior	3	6	−	−	+	−	+	−
9	L	68	F	decreased VA	temporal-inferior	2	7	−	−	+	−	+	−
10	R	70	M	decreased VA	temporal-inferior	3	5	+	−	+	+	−	+
11	R	35	M	metamorphopsia	temporal-superior	1	2	−	−	+	+	−	+
12	R	48	M	metamorphopsia	temporal-inferior	2	4	+	−	+	−	−	−

R; right; L, left; M, male; F, female; DD, disk diameter; PVD, posterior vitreous detachment; MH, macular hole; ERM, epiretinal membrane; VH, vitreous hemorrhage, RD, photocoagulation; cryo, cryopexy; M, month.

**Table 2:** Surgical outcomes of patients with idiopathic vasoproliferative tumors.

Case	Side	Follow Up Duration (Month)	Baseline BCVA	Refractive Error (D)	Treatment	BCVA-1M	BCVA-3M	BCVA-6M	BCVA-Final
1	L	12	0.1	−4.25	PC	0.3	0.4	0.8	0.8
2	R	14	0.4	−0.75	PC	1.0	1.0	1.2	1.2
3	L	27	0.3	0.5	PC, cryo	0.5	0.5	0.6	0.7
4	R	12	0.8	2.25	PC, dia	1.0	1.0	0.8	1.0
5	R	14	0.6	−9	PC	1.2	1.2	1.2	1.2
6	L	21	0.8	−3	PC, cryo	0.7	0.5	0.7	1.0
7	R	84	0.9	−2.75	PC, cryo, dia	1.0	1.2	1.2	1.2
8	R	12	0.8	0	PC	0.9	1.0	1.0	1.0
8	L	12	1.0	0.25	PC	1.0	1.0	1.0	1.0
9	L	121	0.01	1.5	PC, cryo	1.0	0.9	1.0	1.0
10	R	12	0.8	2.25	PC	1.2	1.2	1.2	1.2
11	R	12	0.02	−2.5	PC	0.1	0.2	0.3	0.2
12	R	20	0.5	0.5	PC	1.0	1.0	1.2	0.9

R; right; L, left; BCVA, best-corrected visual acuity (decimal); D; dioptor, PC, photocoagulation; cryo, cryopexy; M, month.

## Data Availability

The data presented in this study are available on request from the corresponding author (MI).
